# Fast protein analysis enabled by high-temperature hydrolysis†

**DOI:** 10.1039/d0sc03237a

**Published:** 2020-09-10

**Authors:** Yuchen Wang, Wenpeng Zhang, Zheng Ouyang

**Affiliations:** State Key Laboratory of Precision Measurement Technology and Instruments, Department of Precision Instrument, Tsinghua University Beijing 100084 P. R. China ouyang@tsinghua.edu.cn zhangwp116@outlook.com

## Abstract

While the bottom-up protein analysis serves as a mainstream method for biological studies, its efficiency is limited by the time-consuming process for enzymatic digestion or hydrolysis as well as the post-digestion treatment prior to mass spectrometry analysis. In this work, we developed an enzyme-free microreaction system for fast and selective hydrolysis of proteins, and a direct analysis of the protein digests was achieved by nanoESI (electrospray ionization) mass spectrometry. Using the microreactor, proteins in aqueous solution could be selectively hydrolyzed at the aspartyl sites within 2 min at high temperatures (∼150 °C). Being free of salts, the protein digest solution could be directly analyzed using a mass spectrometer with nanoESI without further purification or post-digestion treatment. This method has been validated for the analysis of a variety of proteins with molecular weights ranging from 8.5 to 67 kDa. With introduction of a reducing agent into the protein solutions, fast cleavage of disulfide bonds was also achieved along with high-temperature hydrolysis, allowing for fast analysis of large proteins such as bovine serum albumin. The high-temperature microreaction system was also used with a miniature mass spectrometer for the determination of highly specific peptides from *Mycobacterium tuberculosis* antigens, showing its potential for point-of-care analysis of protein biomarkers.

## Abstract 2

A high-temperature microreaction system is developed for fast and selective hydrolysis of proteins, enabling direct analysis of protein biomarkers by mass spectrometry.

## Graphical abstract

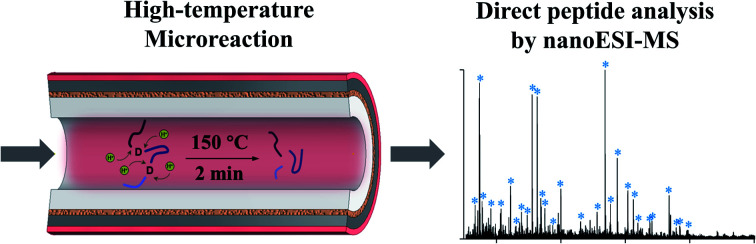

## Introduction

Mass spectrometry (MS) has become a powerful tool for identification of protein primary sequences, post-translational modifications (PTMs) and protein–protein interactions; it also enables determination of various cellular functions at the protein level.^1^ Although an enormous effort has been put into proteomic studies and the potential of protein biomarker identification for clinical diagnosis or therapeutic treatment, currently MS-based protein analysis procedures are still time-consuming and labor intensive, which are not compatible with potential clinical or point-of-care (POC) applications. Bottom-up proteomics^2^ is one the most widely used strategies for protein analysis, which usually requires digestion of proteins into peptides by proteases with high sequencing selectivity.^3–5^ For example, trypsin is widely used for breaking at the C-terminal side of lysine or arginine of peptides under simulative physiological conditions. Most protease-based digestion methods, however, require long-time (∼hours) incubation.^6^ To accelerate the incubation process, approaches such as microwave-assisted trypsin digestion^7,8^ and immobilization of trypsin into different materials^9,10^ have been explored, enabling sufficient trypsin digestion in much shorter time periods.

The enzymatic digestion processes, along with the following enrichment processes, typically require high concentrations of salts in the protein solution, which are not compatible with liquid chromatography (LC)-MS analysis. Extensive sample purification is required to avoid the loss in efficiency for both separation and electrospray ionization (ESI). For this reason, matrix-assisted laser desorption ionization (MALDI)^11,12^ is widely used for MS analysis of digested peptides, which is less sensitive to the matrix effect due to the salts in the samples. However, LC-MS analysis using ESI, which requires sample pretreatment, still plays a major role in proteomic studies, due to better sensitivity, quantitative precision, and sequence coverage obtained with MS/MS analysis of ions at higher charge states.^13,14^ Although some efforts have been made to accelerate the process of enzymatic digestion, ESI-MS analysis would still be slowed down by the time-consuming purification and post-digestion sample treatment procedures.

It is highly desirable to have fast and simple procedures developed for MS-based protein analysis, which would be important not only for proteomic research but also for applying protein analysis in clinical diagnosis.^15–19^ In this work, we developed a method for selective protein hydrolysis with high efficiency without involving enzymatic digestion with high-concentration salt solutions. A simple procedure was developed using only a weak acid and fast incubation at high temperatures. Protein hydrolysis under strong acidic conditions has been previously used for analysis of proteins and peptides.^20,21^ It does not involve high-concentration salts in solution but is usually used to recover total amino acids since the sequence information would be lost due to the nonspecific cleavage of peptide bonds.^22^ Using weak organic acids, selective cleavage at aspartyl residues (Asp, D) in proteins or peptides was achieved.^23,24^ This process was shown to benefit from high temperatures for incubation but still took two hours to obtain optimal results. Cooper *et al.*^25,26^ developed a similar strategy (subcritical water hydrolysis) but without using acids. The hydrolysis process was accelerated by placing the protein solution into a stainless-steel tubing and applying higher temperatures (160 to 207 °C) with an oven. Higher recovery was obtained for model proteins; however, this hydrolysis method was less selective for the breakdown of peptide bonds. These reports testified the effectiveness of the water solution-based high-temperature hydrolysis method for the sequencing of proteins. However, the overall time of the protein analysis is still relatively long (>20 min) due to the requirement of sample pretreatment, pre-conditioning, post-treatment, and multiple-step sample transfer. Recently, in a study of ESI, Chen *et al.* applied ultrahigh temperatures and high pressures for desolvation and reported the fragmentation of protein and peptide ions.^27,28^ Inspired by this observation, we developed an in-solution protein hydrolysis method with superheating in a time shorter than 2 min. Protein sequence information was well recovered with the efficient and highly specific cleavage at Asp. Using nanoESI-MS, this method has been validated for the analysis of standard proteins such as ubiquitin. The application for phosphoprotein and glycoprotein analyses has also been explored. By incorporating a reducing agent into the protein solutions, disulfide bonds could also be cleaved along with fast hydrolysis, allowing for analysis of large proteins such as bovine serum albumin. The procedures for protein treatment and analysis were significantly simplified by using a high-temperature microreaction system. The integrated microreaction system is of low cost and requires low power; this can be important for further transferring it into POC applications. The potential of the microreaction system for biomarker analysis in clinical applications was also demonstrated using a miniature mass spectrometer for MS analysis, determining two antigens of *Mycobacterium tuberculosis* infection, a 10 kDa culture filtrate protein (CFP-10) and a 6 kDa early secretory antigenic target (ESAT-6).

## Results and discussion

### Microreactor for selective protein hydrolysis

**Fig. 1:**
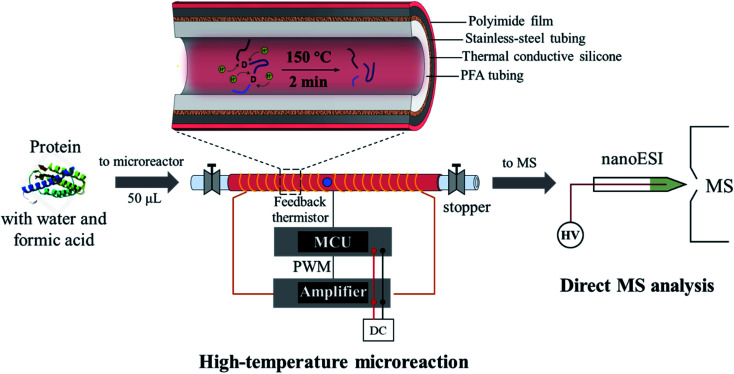
High-temperature microreaction system for fast protein hydrolysis and MS analysis. The heating process was controlled by using a microcontroller unit (MCU), which adjusted the power output from the amplifier through pulse width modulation (PWM).

As shown in Fig. 1, the microreactor used a perfluoroalkoxy (PFA, o.d. 1/16 inch, i.d. 0.02 inch) tubing for loading the sample, which was sheathed with a stainless-steel tubing to improve heat transfer. A relatively low power (12 V) electric heating module was fabricated. A coiled alloy heating wire was used to heat the tubing to a high temperature (*e.g.* 150 °C) within 20 s (Fig. S1†). In a typical procedure, a protein solution prepared with water and weak acid (*e.g.* formic acid) was loaded into the PFA tubing using a syringe; both ends of the tubing were then sealed by using two removable stoppers and the heating was performed for less than 2 min; after cooling in air for 30–60 s, the protein solution was transferred into a glass capillary (i.d. 0.8 mm) with a pulled tip in an offline fashion for subsequent nanoESI-MS analysis. For high-temperature reaction, although this method avoided applying high pressures on the protein aqueous solution, a high-pressure environment may still be created since a temperature over the boiling point of water at atmospheric pressure was applied (typically at 150–180 °C). Fast and selective cleavage of aspartyl sites (X-Asp and Asp-X) of proteins or peptides could be achieved under the high-temperature and high-pressure conditions (Fig. S2†).

**Fig. 2:**
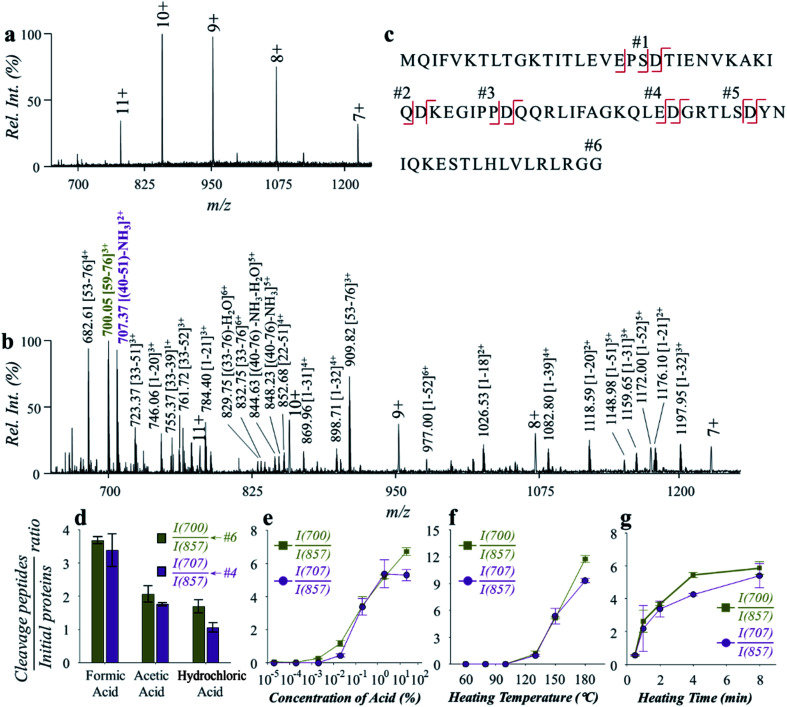
Fast analysis of ubiquitin by high-temperature hydrolysis and nanoESI-MS. Spectra of (a) ubiquitin before hydrolysis and (b) after hydrolysis by the microreaction system. (c) Sequence of ubiquitin and cleavage sites by hydrolysis. Effects of (d) acid types, (e) concentration of formic acid, (f) heating temperature and (g) heating time on hydrolysis efficiency toward ubiquitin. Ubiquitin aqueous solution (0.12 mg mL^−1^) of 50 μL was used for the investigation of the effect of each condition. Formic acid of 2% (v/v), temperature of 150 °C and time of 2 min were used unless otherwise specified. Each value in (d)–(g) represents the mean ± s.d. (*N* = 3).

Hydrolysis of ubiquitin (76 amino acids, with 5 aspartyl sites, MW: 8.56 kDa) was performed to evaluate the effectiveness and efficiency of the microreaction system. Ubiquitin aqueous solution (10 μM, with 2% formic acid, v/v) of 50 μL was prepared for demonstration, which was hydrolyzed at 150 °C for 2 min. Analysis was performed by nanoESI-MS in the positive ion mode on a quadrupole time-of-flight (Q-TOF) mass spectrometer. As shown in Fig. 2, ubiquitin ions were observed at multiple charge states of +7, +8, +9, +10 and +11 in the positive ion mode before hydrolysis (Fig. 2a). After hydrolysis, a large number of peptide peaks were observed in the range of *m*/*z* 650–1250 (Fig. 2b), suggesting the breakdown of ubiquitin by the high-temperature reaction. These peptides were attributed to the cleavage of the peptide chains at the X-Asp and Asp-X sites. As expected, cleavages of all of five Asp sites were observed (Fig. 2a, #1: MQIFVKTLTGKTITLEVEPS; #2: TIENVKAKIQ; #3: KEGIPP; #4: QQRLIFAGKQLE; #5: GRTLS; #6: YNIQKESTLHLVLRLRGG), which can be used for identification of the amino acid sequences of proteins. The loss of H_2_O from glutamic acid (Glu) and loss of NH_3_ from glutamine (Gln) at the N-terminal were commonly observed during hydrolysis of proteins.^29^ This was also observed in the high-temperature hydrolysis method, for example, #4 peptide (QQRLIFAGKQLE, 1429.80 Da) was observed at *m*/*z* 707.37 (2+), with a mass loss of 17.06 Da (–NH_3_). Using a Q-TOF instrument, the peptides can be identified by both the accurate mass and MS/MS experiments, as shown in Fig. S3.† It should be noted that no obvious peptide peaks from oxidation or rearrangement were observed after high-temperature hydrolysis, which is beneficial for the identification of peptides.

The impact of experimental conditions such as the temperature, heating time and solvent compositions was investigated using ubiquitin in aqueous solution (0.12 mg mL^−1^). Weak organic acids such as formic acid and acetic acid were reported to exhibit selective hydrolysis of proteins at the sites of X-Asp and Asp-X, while strong inorganic acids such as hydrochloric acid and sulfuric acid were often used for a more complete, but less selective hydrolysis of proteins.^30,31^ We compared the use of different types of acids, including formic acid, acetic acid, and hydrochloric acid. The pH values of the aqueous solutions were adjusted to 2.5, and the reaction was performed under 150 °C for 2 min. As shown in Fig. 2d, hydrolysis of ubiquitin was observed using either of the acids. It is worth noting that hydrolysis with hydrochloric acid was also shown to have a selectivity for cleavage at the aspartyl sites. By monitoring the intensity ratio between YNIQKESTLHLVLRLRGG (*m*/*z* 700.05), QQRLIFAGKQLE (*m*/*z* 707.37) and the +10 peak of ubiquitin (*m*/*z* 857.33), formic acid was found to have higher hydrolysis efficiency. Presumably this was due to a higher concentration of formic acid in aqueous solution of the same pH value, when comparing with acetic acid and hydrochloric acid. Since the dissociation of formic acid in the aqueous solution is an endothermic reaction, more H^+^ would be produced at high temperatures. This would benefit the hydrolysis reaction that is catalyzed by H^+^ in aqueous solution. In a further test on the effect by acid concentration, the efficiency of ubiquitin hydrolysis increased dramatically with the increase of formic acid contents from 0.02% to 2% (v/v) (Fig. 2e). Although further increase of acid concentration can also improve ubiquitin hydrolysis to some degree, direct ESI of the hydrolyzed peptide solutions would be interfered by high content of acids. Use of formic acid concentration at 2% (v/v) showed a good balance between the efficiencies of hydrolysis and direct ESI or nanoESI for MS analysis.

The effect of temperature was investigated in the range of 60–180 °C. As shown in Fig. 2f, the hydrolysis efficiency increased significantly when temperatures over 130 °C were applied. However, unselective cleavage of peptide bonds was observed at higher temperatures above 180 °C. Temperatures between 130 and 180 °C can be used to obtain high-quality mass spectra of peptides for protein structure identification and quantitative analysis. For further evaluation and application, a hydrolysis temperature around 150 °C was used in consideration of both high hydrolysis efficiency and high selectivity. The effect of heating time was also investigated over a range of 0.5–8 min. As shown in Fig. 2g, obvious protein hydrolysis was observed with heating longer than 0.5 min, and time of 2–5 min were adequate to obtain good hydrolysis efficiencies for fast protein analysis.

The peptide distribution of ubiquitin obtained by the high-temperature hydrolysis and nanoESI-MS was compared with the theoretical peptide distribution of ubiquitin through breakdown at the aspartyl site. As shown in Fig. S4,† all the peptides detected were found to be associated with the breakdown of Asp-X or X-Asp. A special peptide (MQIFVKTLTGKTITLEVE) was observed with loss of two amino acids. Although peptides with different aspartyl sites were not at equal percentages, the results indicated a high selectivity for peptide breakdown at the aspartyl site. As the protein hydrolysis was performed inside the microreactor, possible loss of materials during reaction or solution transfer could affect the analytical throughput and efficiency of peptide quantitation by MS. Using chemically inert PFA tubing as the container of the protein solution for reaction can help to reduce the adsorption of proteins or peptides during the transfer of the solution and to avoid side reactions between proteins and tubing materials. As to the reaction process, the high-temperature hydrolysis with formic acid showed a high selectivity for the breakdown of proteins at the aspartyl site. Therefore, the microreaction system should be able to provide high material recovery for protein sequencing or quantitative analysis.

The microreaction system was also applicable to proteins of relatively low concentrations. For example, a good limit of detection at 0.44 μg mL^−1^ (at a signal-to-noise ratio of 3) was obtained for the analysis of ubiquitin. Besides nanoESI-MS, the fast hydrolysis method can also be coupled with LC-ESI-MS for protein analysis. For example, the peptides hydrolyzed from ubiquitin were well separated by reverse-phase LC and detected by high-resolution MS (Fig. S5†). Although the high-temperature microreaction system was designed for fast analysis of proteins for potential biomedical applications, its coupling with LC-MS could be an alternative for comprehensive bottom-up proteomic analysis.

In comparison with the regular trypsin digestion method, the high-temperature hydrolysis method showed a comparable capability in terms of selective breakdown of low-abundance proteins and peptide coverages. This would make it applicable to bottom-up protein analysis. The high-temperature hydrolysis method avoided using salts for protein digestion, which can simplify sample post-treatment and enable direct analysis of peptide digests by ESI-based methods, such as nanoESI-MS and LC-ESI-MS (Table S1†). More importantly, the time for sufficient protein digestion was significantly reduced to 2–5 min. By incorporating the process using the microreaction system, it is convenient to transfer it to different applications, for example, coupling with the miniature MS system for in-field or POC analysis.

### Fast analysis of proteins with disulfide bonds

Linking through disulfide bonds (S–S) between two cysteine residues is a common type of post-translational modification in proteins during protein biosynthesis inside the cell.^32^ The cleavage of the disulfide bonds is usually highly desirable for bottom-up protein analysis to obtain better coverage of the peptide sequences.^33,34^ Reduction of disulfide bonds can be achieved using reagents such as dithiothreitol (DTT) and tris(2-carboxyethyl)phosphine (TCEP), which can break the disulfide bonds groups in proteins or peptides. The reduction is usually performed along with protease-based digestion and requires 0.5–1 h for incubation and reaction. In this work, an attempt was made to incorporate the reduction of disulfide bonds into the protein hydrolysis process.

**Fig. 3:**
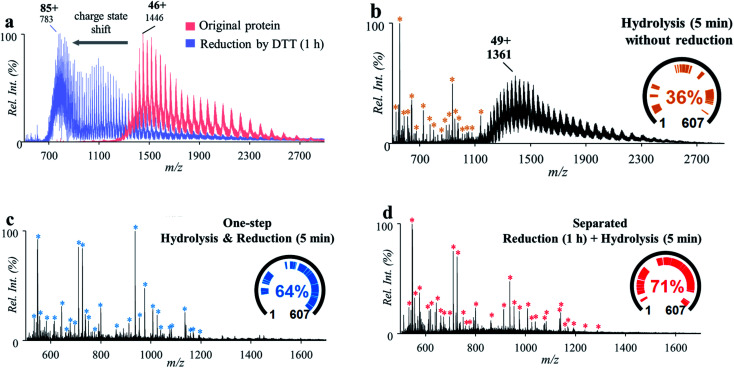
(a) Mass spectra of BSA before (red) and after (blue) reduction with DTT for 1 h. (b) Mass spectrum of BSA after high-temperature hydrolysis without reduction. (c) Mass spectrum of BSA treated by separated procedures: reduction by incubation with DTT for 1 h and then high-temperature hydrolysis for 5 min. (d) Mass spectrum of BSA by one-step high-temperature hydrolysis and reduction for 5 min. Peptides identified by MASCOT were marked with * and listed in Tables S2–S4.†

A series of experiments were performed for this investigation with bovine serum albumin (BSA, MW: 67 kDa; 3 μM, 2% formic acid, v/v), which has 17 disulfide bonds and 40 aspartyl sites. Treatment of BSA with a standard reduction process (12 mM DTT for 1 h under 50 °C) showed a significant charge shift (*e.g.*, from +46 to +85) of the MS spectra of BSA in the positive ion mode (Fig. 3a), indicating the breakdown of the disulfide bonds within the protein structure. After treatment of the original BSA solution by using the microreaction system (150 °C for 5 min), some peptide peaks were observed in the range of *m*/*z* 500–1200 (546.30–4872.49 Da), showing cleavage of proteins at aspartyl sites (Fig. 3b). However, due to the presence of disulfide bonds, a high-abundance group of molecular peaks were also observed, with molecular weights larger than 60 kDa. In another experiment, the BSA solution was first treated with DTT (12 mM) for the reduction of disulfide bonds (incubation at 50 °C for 1 h) and then injected into the microreactor for high-temperature hydrolysis (150 °C for 5 min). As shown in Fig. 3c, abundant peptide peaks were also detected in the range of *m*/*z* 500–1200 (546.30–6429.30 Da), while no obvious peaks of intact proteins were observed. This showed that complete cleavage of peptides of BSA was achieved by combination of separated procedures of reduction and hydrolysis. To prevent possible re-formation of disulfide bonds, 2-iodoacetamide (IAA, 60 mM) was added after reduction by DTT. As shown in Fig. S6,† no significant difference in the peptide recovery was observed after treatment by IAA.

To accelerate the reduction of disulfide bonds, DTT (60 mM) was introduced into the BSA solution (3 μM, 2% formic acid, v/v) and then the mixture was treated using the microreaction system (150 °C). IAA was not used in order to simplify the sample preparation procedures. It was interesting that after reaction for 5 min, abundant peptide peaks were also detected in the range of *m*/*z* 500–1200 (546.30–5341.56 Da), while no obvious peaks of intact proteins were observed (Fig. 3d). This result showed that the cleavage of both peptide bonds and disulfide bonds was simultaneously achieved by this one-step approach. The cleavage of disulfide bonds may have been accelerated through increasing the accessible space of the protein structure at higher temperatures. Furthermore, the cleavage of some peptide bonds at aspartyl sites could also improve the contact of DTT with protein disulfide bonds. Although DTT is usually used under basic conditions, the incorporation of DTT into formic acid solution showed a good reduction efficiency for BAS analysis using the proposed system. This was also effective for a small peptide (oxytocin, MW: 1007.19 Da), which has a disulfide bond but no aspartyl site. As shown in Fig. S7,† reduction yield of 72% was obtained with formic acid (2%, v/v) by the high-temperature reaction (150 °C, 5 min). Reduction of oxytocin and BSA was also performed by using TECP as the reducing agent. Although higher reduction yield was obtained for oxytocin (Fig. S7†) with TECP, the overall peptide coverage of BSA obtained with TECP (Fig. S8†) was just comparable to that obtained with DTT. These results showed that it is possible to apply the high-temperature microreaction system to analysis of complicated proteins using a simple composition of solution and simplified procedures.

### Analysis of proteins with PTMs

**Fig. 4:**
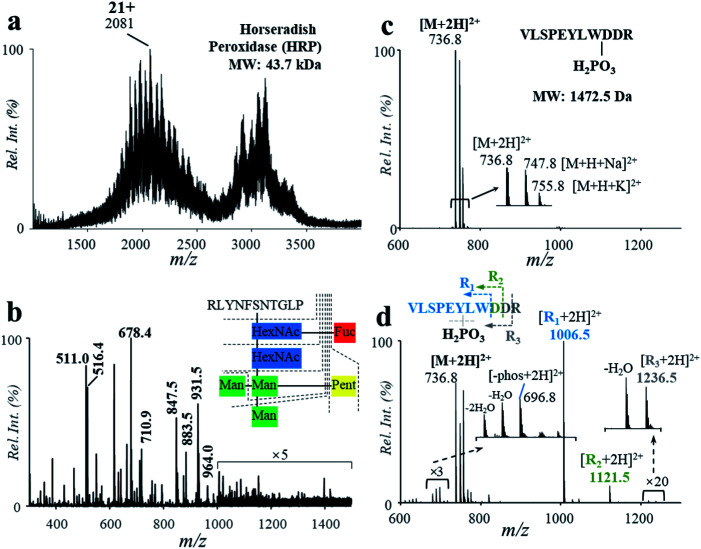
Mass spectra of HRP (a) before and (b) after high-temperature hydrolysis. Mass spectra of the phosphopeptide (c) before and (d) after high-temperature hydrolysis.

PTMs play a crucial role in protein structuring and functioning. PTMs were commonly observed in proteins, which need to be analyzed for the identification of proteins. In this study, a glycoprotein and a phosphopeptide were used to investigate the influence of the acidic, high-temperature and high-pressure solvent conditions on the stability of glycosyl and phosphate groups during the protein hydrolysis. Horseradish peroxidase (HRP, MW: 43.7 kDa) was used for demonstration, which consists of 308 amino acid units, 21 aspartyl sites and a number of *N*-glycosylated sites.^7^ Using solutions of HRP (5.8 μM, 2% formic acid, v/v), relatively high hydrolysis efficiency was obtained by the high-temperature reaction, with the peaks of intact protein (*m*/*z* 2081, 21+) decreased significantly after hydrolysis (Fig. 4a and b). Highly abundant peaks of peptides were observed in the range of *m*/*z* 300–1500 in the positive ion mode (Fig. 4b). In comparison with the results in the PER1A_ARMRU sequence from the SwissProt Database (https://www.uniprot.org/uniprot/P00433), 59% of the sequence can be identified (Fig. S9†). Besides, some peaks were observed at a relative low intensity in the range of *m*/*z* 1000–1500, such as *m*/*z* 1006.5, *m*/*z* 1087.5, and *m*/*z* 1153.5. In comparison with data reported from the literature^7^ and SwissProt Database (https://www.uniprot.org/uniprot/P00433), these peaks should be glycopeptides (Table S5†) after cleavages at fucose (Fuc), mannose (Man), *N*-acetylhexosamine (HexNAc), and pentose (Pent) (Fig. S10†).

The influence of the high-temperature reaction on the phosphorylation groups was also investigated by using a peptide with a gamma-phosphate on the hydroxyl group of tyrosine (VLSPE-pY-LWDDR; MW: 1472.5 Da). A solution of the phosphopeptide (43 μM, 2% formic acid, v/v) was analyzed using the high-temperature hydrolysis followed by nanoESI-MS analysis. As shown in Fig. 4c and d, a doubly charged peak was observed at *m*/*z* 736.8 ([M + 2H]^2+^) with a high intensity. Due to the presence of Na^+^ and K^+^ in the phosphopeptide sample, peaks of *m*/*z* 747.8 ([M + H + Na]^2+^) and *m*/*z* 755.8 ([M + H + K]^2+^) were also obtained. After the high-temperature hydrolysis, several singly charged peaks were clearly observed in the range of *m*/*z* 1000–1300. The peak at *m*/*z* 1211.5 was identified as the peptide residual from cleavage of an aspartyl at the C-terminal and the phosphate group, while *m*/*z* 1006.5 was identified for cleavage of both aspartyl sites and the phosphate group (VLSPEYLW, confirmed by MS/MS, Fig. S11†). These results show that although PTMs such as glycation and phosphorylation were mostly cleaved during the high-temperature hydrolysis, other peptide residuals obtained could be used to indicate the PTMs.

### Application in the analysis of protein biomarkers of *Mycobacterium tuberculosis*

Fast determination of protein biomarkers is significant for clinical analysis and disease diagnosis.^35^ For many protein biomarkers, their identification might only require the detection of presence of intact proteins, which can be realized by immunoaffinity methods. For many other protein biomarkers, however, the identification would require confirmation of certain peptide sequences for the purpose of accurate diagnosis of diseases. For example, CFP-10 and ESAT-6 are two biomarkers of *Mycobacterium tuberculosis* (*Mtb*) infections for the diagnosis of tuberculosis (TB) disease.^36^ The detection of intact CFP-10 and ESAT-6 using immunoaffinity methods can cause false positive results in TB diagnosis, since some nontuberculous mycobacteria can also secrete similar proteins.^37^ Comprehensively screening peptide sequences has revealed that some peptides of high *Mtb* specificity, such as the tryptic digests of CFP-10 (TDAATLAQEAGNFER) and ESAT-6 (WDATATELNNALQNLAR), can be used for accurate diagnosis of TB.^38^

**Fig. 5:**
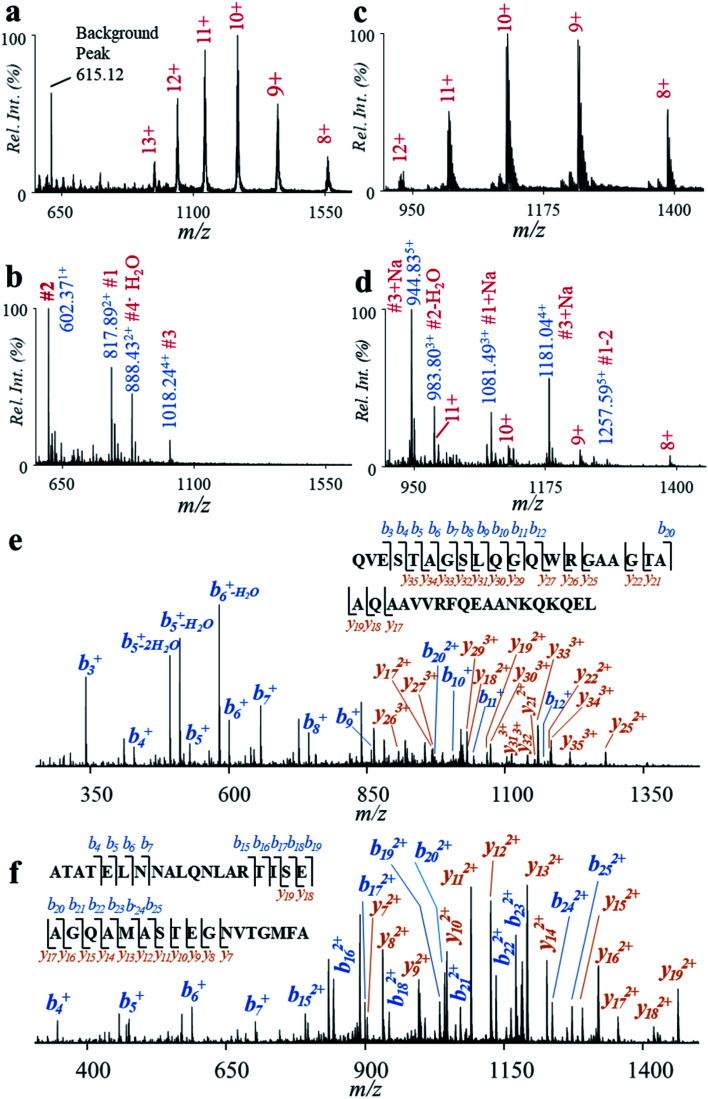
Mass spectra of CFP-10 (a) before and (b) after hydrolysis. Mass spectra of ESAT-6 (c) before and (d) after hydrolysis. MS/MS spectra of (e) peptide #3 hydrolyzed from CFP-10 and (f) peptide #3 hydrolyzed from ESAT-6.

In this study, we explored the fast analysis of peptides from CFP-10 and ESAT-6 by the high-temperature hydrolysis and nanoESI-MS method. Recombinant CFP-10 (MW: 11.73 kDa) and recombinant ESAT-6 (MW: 11.11 kDa) were used. CFP-10 in formic acid solution was detected with +8 to +13 charges by using nanoESI and Q-TOF (Fig. 5a). As shown in Fig. 5b, the peaks of CFP-10 almost disappeared after reaction at 150 °C for 2 min, while peptide peaks were well detected in the range of *m*/*z* 550–1100 in the positive ion mode. Four typical peptides from the hydrolysis of four aspartyl sites were well detected (Table 1) at *m*/*z* 817.89 (#1, AATLAQEAGNFERISG), *m*/*z* 602.37 (#2, LKTQI), *m*/*z* 1018.24 (#3, QVESTAGSLQGQWRGAAGTAAQAAVVRFQEAANKQKQEL), and *m*/*z* 888.43 (#4, EISTNIRQAGVQYSRA). Good hydrolysis efficiency was also obtained for recombinant ESAT-6 (Fig. 5c and d). Three typical peptides from the hydrolysis of two aspartyl sites were well detected (Table 1) at *m*/*z* 1081.49 (#1, MTEQQWNFAGIEAAASAIQGNVTSIHSLL, with N-terminal tag), *m*/*z* 983.80 (#2, EGKQSLTKLAAAWGGSGSEAYQGVQQKW), and *m*/*z* 944.83 and *m*/*z* 1181.04 (#3, ATATELNNALQNLARTISEAGQAMASTEGNVTGMFA, with a C-terminal tag).

**Table 1:** Hydrolyzed residues of CFP-10 and ESAT-6

Protein	No.	MW (Da)	Charge	Sequence
CFP-10	#1	1633.80	1+	AATLAQEAGNFERISG
	#2	601.38	1+	LKTQI
	#3	4084.08	4+	QVESTAGSLQGQWRGAAGTAAQAAVVRFQEAANKQKQEL
	#4	1791.92	2+	EISTNIRQAGVQYSRA
ESAT-6	#1	3086.51	3+	MTEQQWNFAGIEAAASAIQGNVTSIHSLL
	#2	2964.47	3+	EGKQSLTKLAAAWGGSGSEAYQGVQQKW
	#3	3652.75	4+, 5+	ATATELNNALQNLARTISEAGQAMASTEGNVTGMFA

To confirm if the peptides obtained from high-selective hydrolysis were specific to *Mtb*, we compared the sequences of CFP-10 and ESAT-6 with related proteins from a series of tuberculous and nontuberculous mycobacteria.^36^ As show in Table 2, the sequence of the peptide #3 from CFP-10 (QVESTAGSLQGQWRGAAGTAAQAAVVRFQEAANKQKQEL, confirmed by MS/MS in the positive ion mode, Fig. 5e) was highly specific to *Mtb* and *M. bovis*, while peptides #1 and #4 can also be found in nontuberculous mycobacteria such as *M. kansasii*, *M. marinum* and *M. ulcerans*.^39^ Although the sequence of the peptide #1 (AATLAQEAGNFERISG; MS/MS spectrum shown in Fig. S12†) overlapped partially with that of the characteristic tryptic digest of CFP-10 (TDAATLAQEAGNFER),^38^ it was less specific to *Mtb*. As to ESAT-6, the sequence of the peptide #3 (EGKQSLTKLAAAWGGSGSEAYQGVQQKW, confirmed by MS/MS in the positive ion mode, Fig. 5f) was highly specific to *Mtb* and *M. bovis*, while peptide #1 or #2 can also be found in nontuberculous mycobacteria such as *M. kansasii*. The sequence of this peptide also overlapped with that of the characteristic tryptic digest of ESAT-6 (WDATATELNNALQNLAR). These results showed that the high-temperature hydrolysis and nanoESI-MS method has the potential to be a useful tool for fast determination of protein biomarkers of TB disease.

**Table 2:** Presence of characteristic peptides in CFP-10 and ESAT-6 from different mycobacteria. Protein sequences were from the UniProtKB database and aligned by using CLUSTAL Omega at http://www.uniprot.org/align/ (Y: found; N: not found)

Species	CFP-10, UniProtKB ID	#1	#3	#4	ESAT-6, UniProtKB ID	#1	#2	#3
*M. tuberculosis*	P9WNK4	**Y**	**Y**	**Y**	P9WNK6	**Y**	**Y**	**Y**
*M. bovis*	X5FC46	**Y**	**Y**	**Y**	P0A565	**Y**	**Y**	**Y**
*M. avium*	X7UCW0	N	N	N	X8AC35	N	N	N
					X7U728	N	N	N
					V7J7P5	N	N	N
*M. intracellulare*	B7T007	N	N	N	X8CAA4	N	N	N
	A0A1A2Q365	N	N	N	H8IQR6	N	N	N
	H8IWA2	N	N	N	A0A1A2QF54	N	N	N
	X8CDV4	N	N	N				
	A0A1A2QAU8	N	N	N				
*M. abscessus*	A0A0U1BA85	N	N	N	B1MAY6	N	N	N
					A0A0U0X8G0	N	N	N
					A0A0U1CBI2	N	N	N
					A0A0U0ZCB5	N	N	N
					A0A0U0ZRD0	N	N	N
*M. kansasii*	X7Z0L9	**Y**	N	N	X7Z193	**Y**	**Y**	N
	X7YF15	**Y**	N	N	U5WQ96	**Y**	**Y**	N
	X7YNU7	**Y**	N	N	X7YBX6	**Y**	**Y**	N
	B5TV86	**Y**	N	N	B5LSL6	**Y**	**Y**	N
					X7YMX1	N	N	N
					B5LSL9	**Y**	**Y**	N
					X7YLI9	N	N	N
*M. xenopi*					X7ZDM4	N	N	N
*M. fortuitum*	A0A100WXY5	N	N	N	A0A117IGW4	N	N	N
	K0VRZ7	N	N	N	K0V925	N	N	N
	A0A0N9Y339	N	N	N	A0A0N9YA91	N	N	N
*M. chelonae*					X8EW25	N	N	N
*M. smegmatis*	A0A0D6FTJ8	N	N	N	A0A0D6FU90	N	N	N
	L8FKZ2	N	N	N	L8FPE8	N	N	N
	A0QNJ5	N	N	N	A0QNJ6	N	N	N
					A0A0S1EEI5	N	N	N
*M. marinum*	B2HJI8	**Y**	N	**Y**	B2HNQ2	N	N	N
	B5TV81	**Y**	N	**Y**	B5A909	N	N	N
	B2HNR7	N	N	N	B2HJI9	N	N	N
					B2HKG0	N	N	N
					B2HJJ0	N	N	N
*M. ulcerans*	B2KWS3	**Y**	N	**Y**	A0A1B4Y9X8	N	N	N
	Q5G542	**Y**	N	N	B2KWS6	N	N	N
					B2KWT0	N	N	N
					A0A1B4Y9M4	N	N	N
*M. szulgai*	A0A1A3R643	N	N	N	B5A908	N	N	N
	B5TV80	N	N	N	A0A1A3R6G2	N	N	N
*M. gordonae*	A0A1A6B951	N	N	N	A0A0Q2X3J1	N	N	N
	A0A0Q2RKC3	N	N	N				

### Potential POC biomarker analysis by coupling with a miniature MS system

Miniaturization of the MS systems is an important component for implementing MS in clinical and POC applications.^40,41^ Previously we had developed several direct sampling methods and miniature MS systems for the fast analysis of drugs, metabolites and lipids.^42–49^ Recently, we also explored the application of miniature mass spectrometers in quantitation of synthetic peptides with molecular weights larger than 1500 Da.^50^ However, due to the sophistication of sample preparation procedures, fast analysis of proteins and digested peptides by miniature MS is still difficult.

**Fig. 6:**
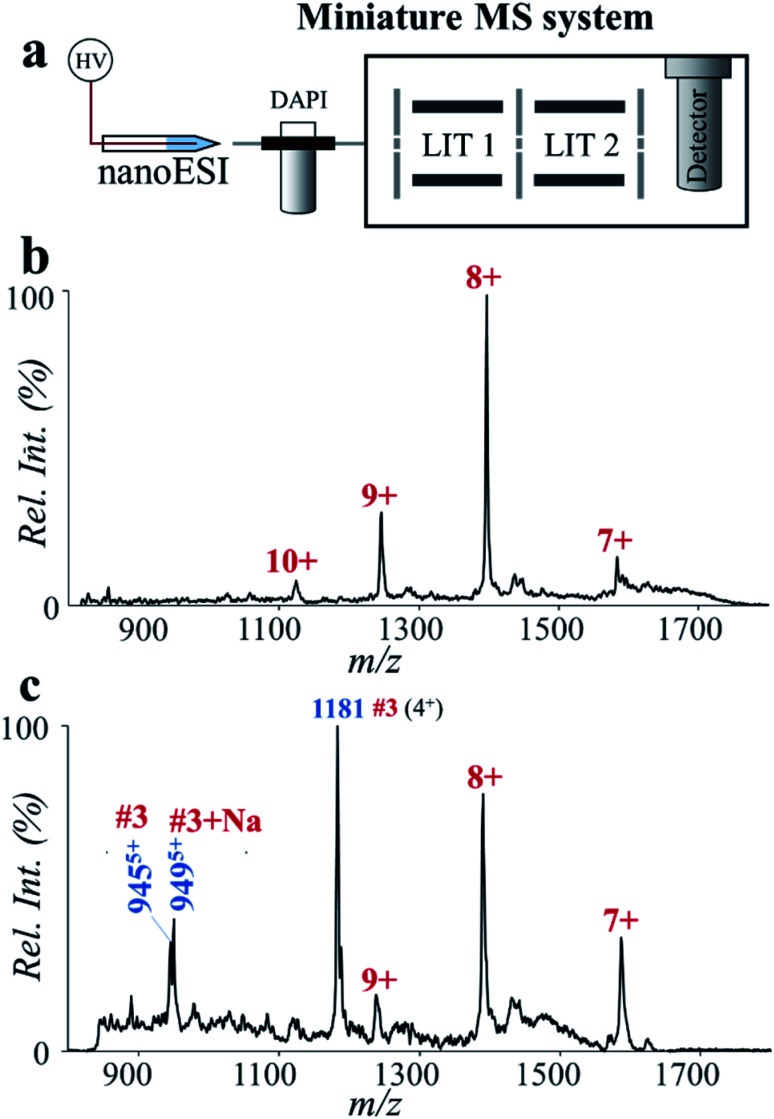
Analysis of ESAT-6 by high-temperature hydrolysis and a miniature MS system. (a) Schematic of the miniature MS system. Mass spectra of ESAT-6 (b) before and (c) after high-temperature hydrolysis, showing fast and sensitive determination of the characteristic peptide highly specific to *Mtb*. ESAT-6 was prepared in water (2% formic acid, v/v) at the concentration of 20 ppm. The solution after treatment with the high-temperature microreaction system (150 °C, 2 min) was directly used for nanoESI-MS analysis.

Here we explored potential POC analysis of protein biomarkers by coupling the high-temperature microreaction system with a miniature dual linear ion trap (LIT) MS system. It was coupled with a nanoESI source, where voltages of 1500–1800 V were used for analysis of proteins and peptides. This miniature MS system was capable of accurate MS analysis up to *m*/*z* 2000,^51^ which enabled detection of major peaks of small proteins such as ESAT-6 (Fig. 6a and b). In comparison with the result by Q-TOF (MS spectrum of ESAT-6, Fig. 5c), an obvious decrease of charge states was observed by using the miniature MS system (Fig. 6a). It probably results from the difference in the ion injection methods. The bench-top Q-TOF used a common continuous injection method while the miniature MS was equipped with a discontinuous atmospheric pressure interface. After high-temperature hydrolysis for 2 min, some major peptide fragments of ESAT-6 were detected by using the nanoESI-based miniature MS system. Due to the difference in ion formation and ion transfer, the mass spectrum of the ESAT-6 peptides collected by using the miniature MS system was slightly different from that obtained by using the Q-TOF instrument. The intensity of peptide #1 (*m*/*z* 1081) was relatively low, but the characteristic peptide (#3, *m*/*z* 944, *m*/*z* 949 and *m*/*z* 1181) was detected at high abundance (Fig. 6c). Although the sensitivity of the overall procedure still needs to be improved for application with clinical samples, determining the characteristic peptides using the fast protein hydrolysis and MS analysis by using the miniature MS system is promising for future TB diagnoses.

## Conclusions

In this work, a microreaction system was developed for the fast and direct analysis of proteins. Proteins were hydrolyzed in the microreactor under high-temperature (∼150 °C) and weakly acidic conditions, enabling selective breakdown of a series of proteins at the aspartyl site in 2 min. Being free of salts, the hydrolyzed solutions from the microreaction system can be directly used for nanoESI-MS analysis. By incorporation of reductants into the solution, one-step cleavage of peptide bonds and disulfide bonds was also realized, allowing for fast analysis of large proteins. The microreaction system is of small size, avoids using high pressures and only requires simple procedures for protein hydrolysis; the coupling of the microreaction system with a miniature mass spectrometer represents a useful tool for POC analysis of protein biomarkers.

## Conflicts of interest

The authors declare no conflicts of interest.

## Supplementary Material

SC-011-D0SC03237A-s001
