# Supplementary material for: Fast protein analysis enabled by high-temperature hydrolysis
Source: Chem Sci. 2020 Sep 10;11(38):10506–16. doi: 10.1039/d0sc03237a (PMC8162451; doi:10.1039/d0sc03237a)
Supplement: SC-011-D0SC03237A-s001 [file SC-011-D0SC03237A-s001.pdf]

Electronic Supplementary Material (ESI) for Chemical Science.
This journal is © The Royal Society of Chemistry 2020

Supporting Information

Fast Protein Analysis Enabled by High-Temperature Hydrolysis

Yuchen Wang[a], Wenpeng Zhang[a,*], Zheng Ouyang[a,*]

[a]State Key Laboratory of Precision Measurement Technology and Instruments, Department of Precision Instruments, Tsinghua University, Beijing 100084, P.R. China

***Corresponding Author:**

E-mail: ouyang@tsinghua.edu.cn (Zheng Ouyang)

zhangwp116@outlook.com (Wenpeng Zhang)

Experimental

Chemicals and Materials. Recombinant ubiquitin (MW: 8.56 kDa) was obtained from UB-biotech (Jilin, China). Bovine serum albumin (BSA, MW: 66.7 kDa) was purchased from Sigma-Aldrich (MO, USA), 11AA-phosphorylated-peptide (VLSPE-pY-LWDDR, MW: 1472 Da) from Guoping Pharmaceutical (Anhui, China), and horseradish peroxidase (HRP, MW: 44.3 kDa) from Acmec Biochemical (Shanghai, China). Recombinant antigens of *Mycobacterium tuberculosis*, including ESAT-6 (MW: 11.11 kDa) and CFP-10 (MW: 11.73 kDa) were purchase from Kitgen Bio-tech (Hangzhou, China). Formic acid (A.R.) was obtained from Fisher Scientific (NH, USA). Other chemicals and reagents were purchased from Sigma-Aldrich (MO, USA). The capillaries with pulled tips for nanoESI were prepared from glass capillaries (o.d. 1.5 mm, i.d. 0.86 mm, Sutter Instrument, CA, USA) using a P-1000 capillary puller (Sutter Instrument, CA, USA)

Fabrication of the high-temperature microreactor. The microreactor was homebuilt as shown in Figure S1a). A PFA tubing (o.d. 1/16 inch, i.d. 0.125 inch) of 10 cm was inserted into a stainless-steel tube (o.d. 2.5 mm, i.d. 2 mm), which was wrapped with an alloy heating wire (Ni80Cr20, o.d. 0.25 mm, 10 Ω) and coated with silicon grease for thermo insulation. A high temperature of 150 °C could be reached in 20 s at 12 V. The inlet and outlet of the PFA tubing were connected with spiral gated valves for fast sealing and opening. The heating process was controlled by a single-chip microcomputer and a thermistor was installed on the stainless-steel tube for temperature feedback. A relatively stable temperature control (±2 °C) was achieved with the control program.

Protein hydrolysis and nanoESI-MS analysis. Protein samples were prepared in purified

water. For analysis, the stock solutions were diluted by water and then mixed with formic acid or acetic acid. The sample solution was injected into the microreactor, sealed by the valves, heated up to 150 °C, and kept for 2-5 min for fast hydrolysis. After protein hydrolysis, the solution was transferred into a glass capillary with a pulled tip for direct analysis by nanoESI-MS. MS experiments were performed on a quadrupole time-of-flight (Q-TOF) mass spectrometer (MaXis Impact, Bruker Daltonics, Bremen, Germany) with a nanoESI source. Mass spectra were converted to MGF format by Bruker Compass DataAnalysis Software (Bruker Daltonics, Bremen, Germany) and subjected to MASCOT searches (Matrix Science Ltd., London, U.K.). Peptide mass tolerance was set as 0.05 Da.

A miniature mass spectrometer (57 cm (length) × 24 cm (width) × 32 cm (height)) was also used for protein and peptide analysis. It was a modified Mini β (PURSPEC Technologies, Beijing, China) instrument, equipped with a discontinuous atmospheric pressure interface (DAPI) for introduction of ions, and two linear ion traps. The mini MS system was coupled with nanoESI , by which voltages of 1500-1800 V were used for analysis of proteins and peptides.

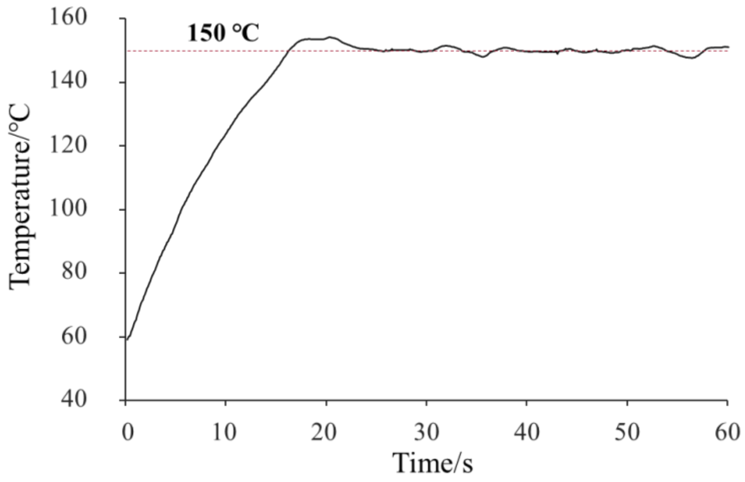

Fig. S1. Change of temperatures detected by the feedback thermistor of the microreactor by setting at 150 °C. It shows that the required temperature reaches within 20 s after heating, and the temperature keeps relatively stable afterwards.

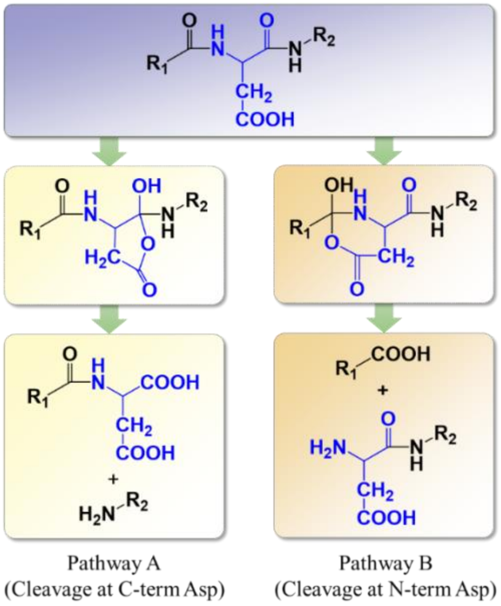

Fig. S2. Mechanism of the selective hydrolysis cleavage at X-Asp and Asp-X sites by high-temperature microreaction. Under acidic condition, an anhydride or a cyclic imide can be formed between the β-carboxyl group and the amide group of the peptide/protein at either terminal. Finally, Asp-X or X-Asp can be cleaved through elimination of one water molecule.[1, 2]

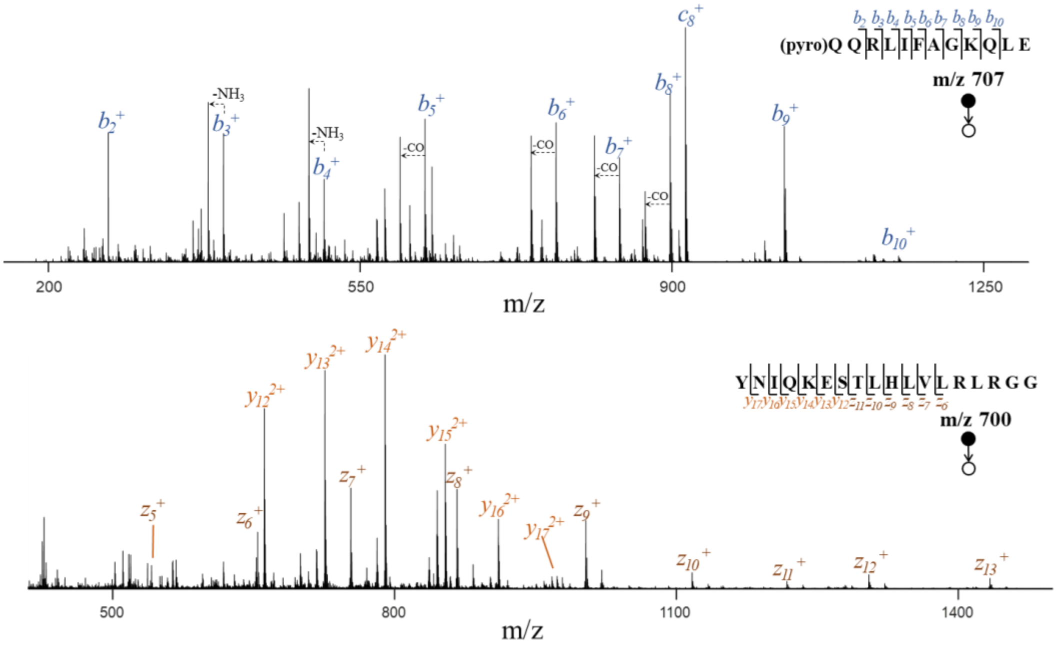

Fig. S3. MS/MS spectra of characteristic peptides hydrolyzed from ubiquitin. The analysis was performed by nanoESI-MS/MS in positive ion mode.

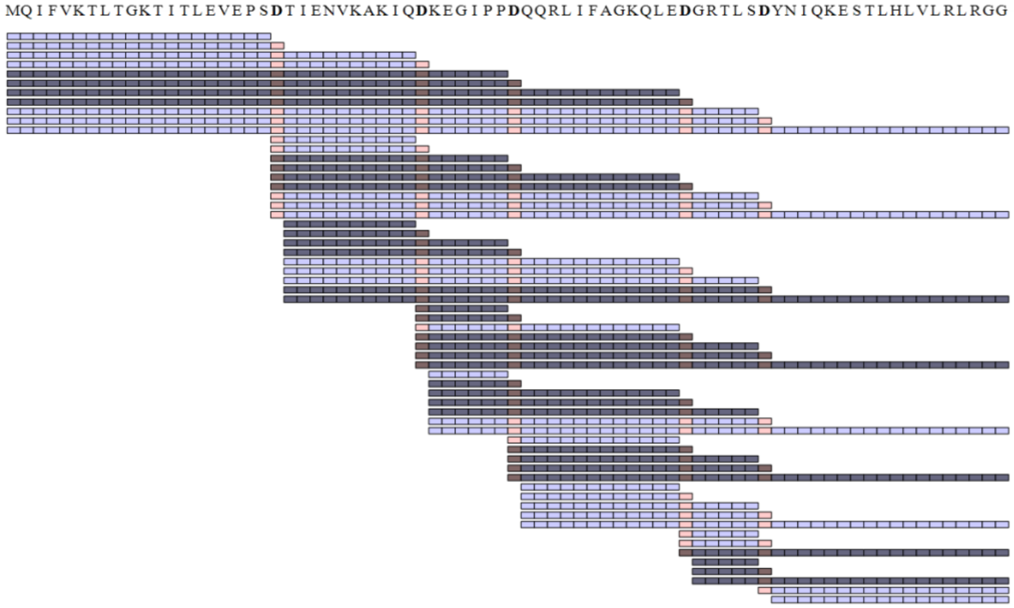

Fig. S4. Theoretical peptide distribution of ubiquitin through breakdown at the aspartyl site. Peptides detected after hydrolysis are labeled in dark grey.

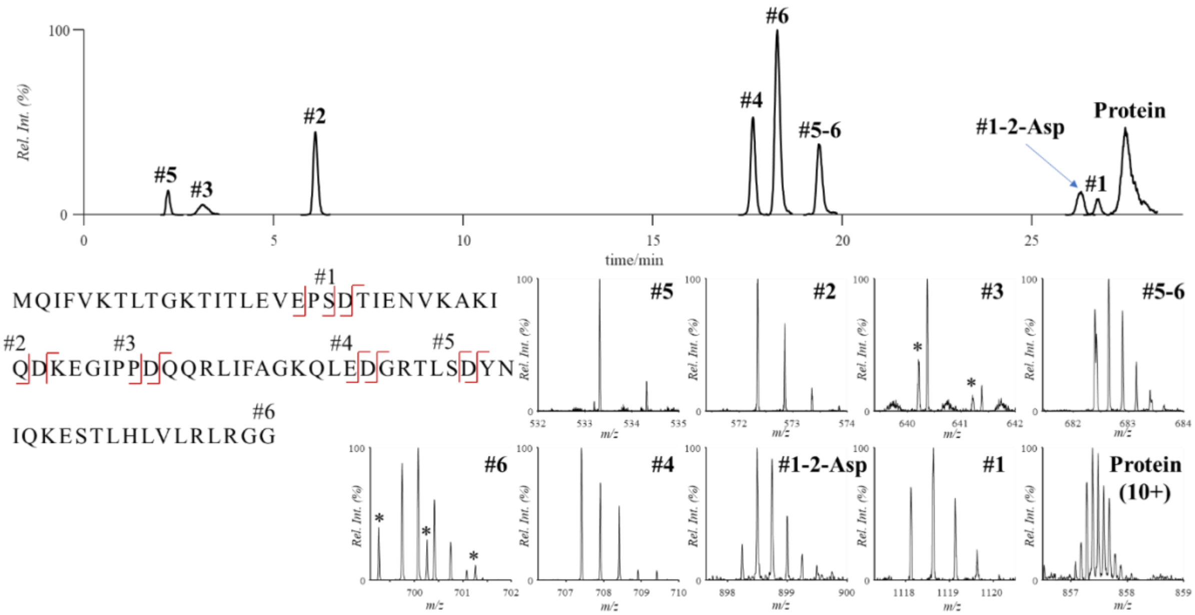

Fig. S5. LC-MS chromatogram and related MS spectra of peptides hydrolyzed from ubiquitin. The analysis was performed by an RPLC-ESI-Q-TOF system in positive ion mode. Impurities were marked with *.

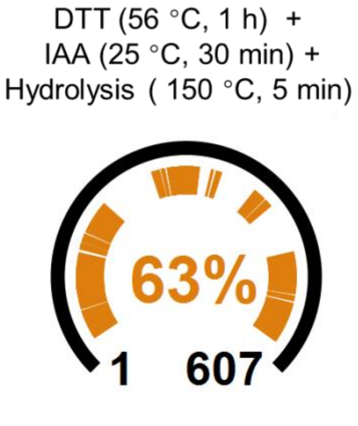

Fig. S6. Recovery of peptides of BSA treated by separated procedures: reduction by incubation with DTT for 1 h, incubation with IAA for 30 min and then high-temperature hydrolysis for 5 min.

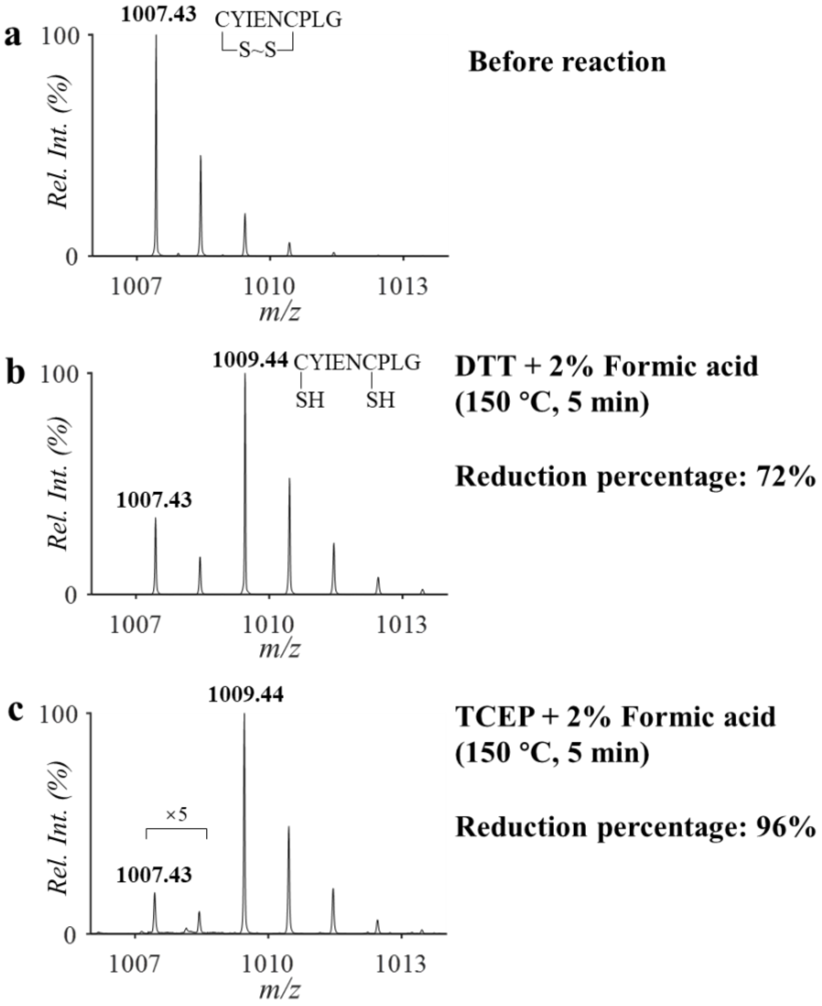

Fig. S7. Mass spectra of oxytocin (a) before reaction, (b) after treatment with DTT (12 mM) for 5 min (150 °C), (c) after treatment of TCEP (12 mM) for 5 min (150 °C). Oxytocin was prepared in water at 20 μM, with 2% formic acid (v/v). nanoESI-MS in positive ion mode was used for analysis. The reduction product was observed at m/z 1009.44, the reduction yield was calculated by the ratio of the reduced intensity of *m/z* 1007.43 to its intensity before reaction.

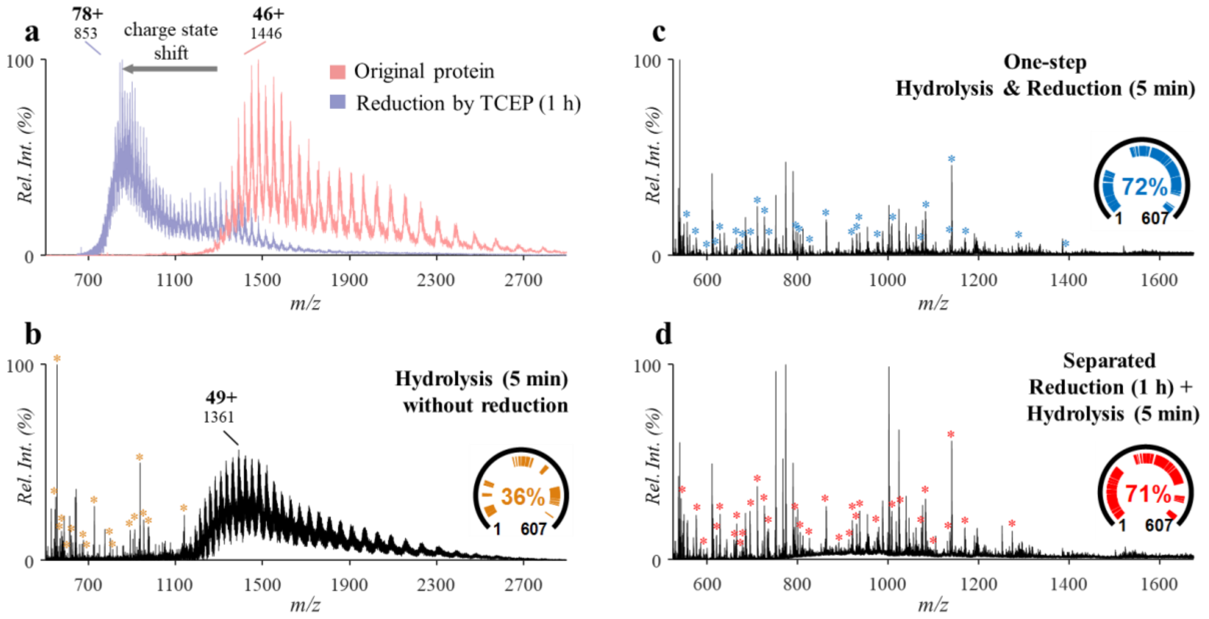

Fig. S8. (a) Mass spectra of BSA before (red) and after (blue) reduction by with TCEP (12 mM) for 1 h. (b) Mass spectrum of BSA after high-temperature hydrolysis without reduction. (c) Mass spectrum of BSA treated by separated procedures: reduction by incubation with TCEP for 1 h and then high-temperature hydrolysis for 5 min. (d) Mass spectrum of BSA by one-step high-temperature hydrolysis and reduction for 5 min. Peptides identified by MASCOT were marked with *.

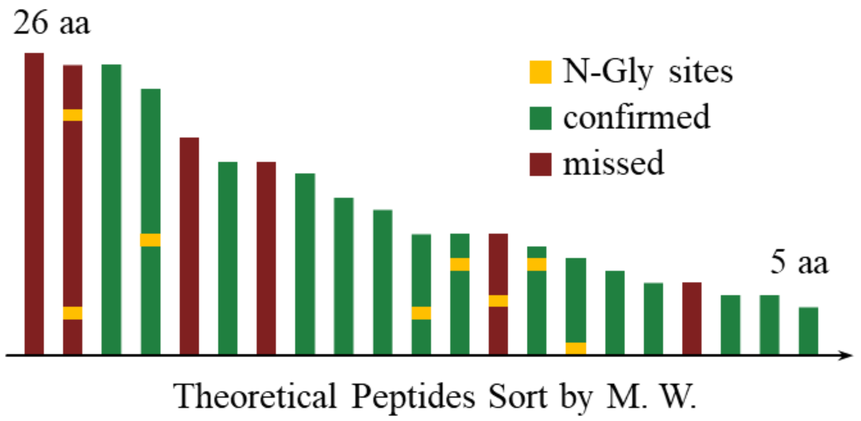

Fig. S9. Amino acid sequence coverage of HRP analyzed by hydrolysis and nanoESI-MS.

Peptides detected after hydrolysis are listed in Table S4.

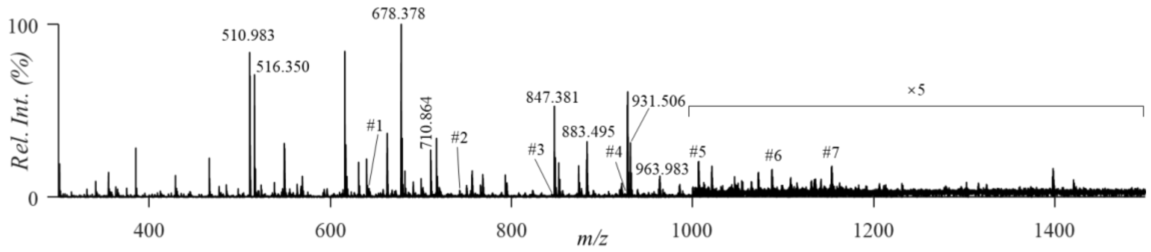

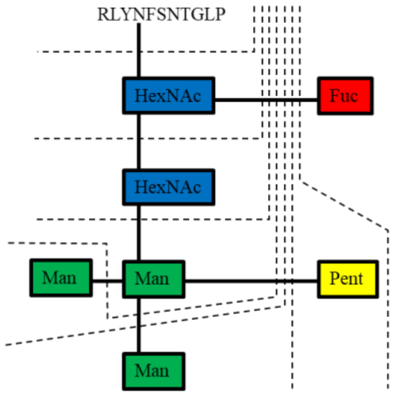

No.	Modifications	Measured m/z	Calculated m/z
**RLYN*FSNTGLP**			
#1	none	641.3342	641.3257(2+)
#2	HexNAc(1)	742.8657	742.8654(2+)
#3	HexNAc(2)	844.4008	844.4051(2+)
#4	HexNAc(2)Man(1)	925.4236	925.4315(2+)
#5	HexNAc(2)Man(2)	1006.446	1006.458(2+)
#6	HexNAc(2)Man(3)	1087.471	1087.484(2+)
#7	HexNAc(2)Man(3)Pent(1)	1153.493	1153.505(2+)
#8	HexNAc(2)Man(3)Pent(1)Fuc(1)	Not Obversed	1226.534(2+)

Fig. S10. Mass spectra and possible cleavage pathways for characterization of glycopeptide peaks from HRP.

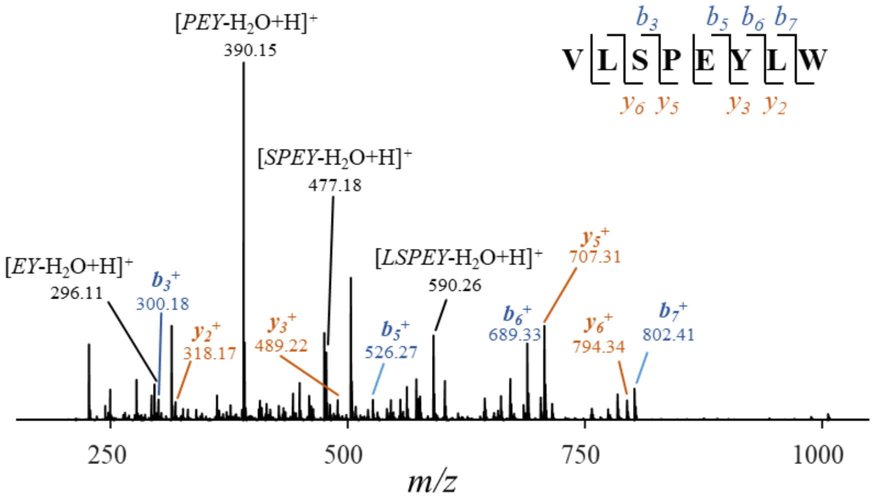

Fig. S11. MS/MS spectrum of peptide residue (VLSPEYLW) after hydrolysis of the phosphopeptide.

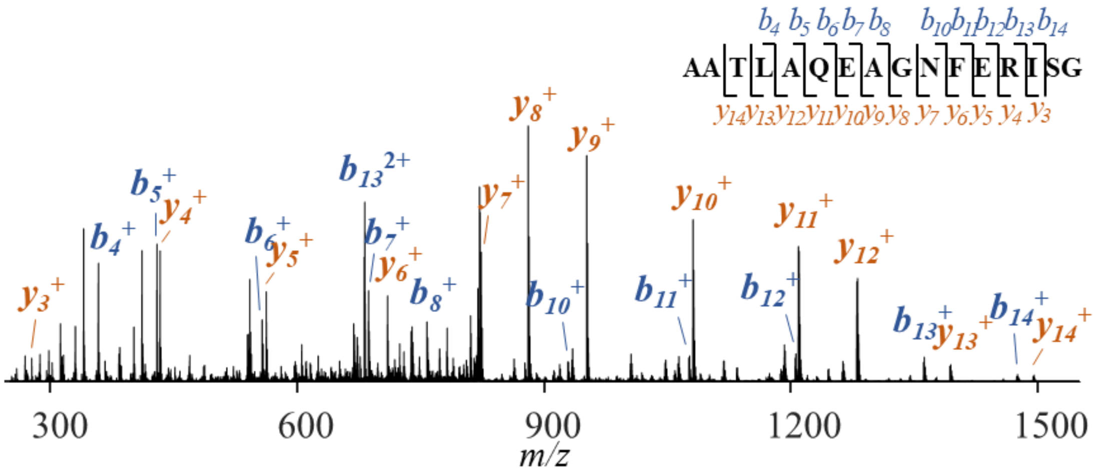

Fig. S12. MS/MS spectrum of peptide #1 (AATLAQEAGNFERISG) hydrolyzed from CFP-10.

Table S1. Comparison of the trypsin-based enzymatic digestion method with the high-temperature hydrolysis method.

	**Trypsin-based enzymatic digestion***	**High-temperature hydrolysis**
Time	~ 12 h	2-5 min
Salt	100 mM ammonium bicarbonate	None
Enzyme	Trypsin	None
Reagent	Urea	Formic acid
Temperature	~ 37 °C	120 °C - 180 °C
Post-treatment	Desalting and purification	None
Analytical methods	LC-MS<br>MALDI-MS	nanoESI-MS<br>LC-MS<br>Miniature MS

* Information from *Nature Biotechnology* 2001, 19, 242–247

Table S2. MASCOT searching results of 2-min BSA high-temperature hydrolysis without DTT.

Start	End	Observed	Mr(expt)	Mr(calc)	Delta	M	Peptide
25	37	1583.8004	1582.7931	1582.7852	0.0079	2	R.DTHKSEIAHRFKD.L
26	36	1353.7428	1352.7355	1352.7313	0.0042	0	D.THKSEIAHRFK.D
26	37	1468.7858	1467.7785	1467.7582	0.0203	1	D.THKSEIAHRFKD.L
38	60	2666.3467	2665.3394	2665.3489	- 0.0094	0	D.LGEEHFKGLVLIAFSQYLQQCPF.D<br>+ Dehydro (C)
38	61	2782.3494	2781.3421	2781.3836	- 0.0415	1	D.LGEEHFKGLVLIAFSQYLQQCPFD.E
97	109	1450.737	1449.7297	1449.7286	0.0011	0	D.ELCKVASLRETYG.D<br>+ Glu->pyro-Glu (N-term E)
135	142	925.5381	924.5308	924.528	0.0028	2	P.DLPKLKPD.P
136	141	695.4837	694.4764	694.4741	0.0023	0	D.LPKLKP.D
136	142	810.5108	809.5035	809.5011	0.0025	1	D.LPKLKPD.P
143	152	1137.5379	1136.5306	1136.5172	0.0134	2	D.PNTLCDEFKA.D
149	152	476.2519	475.2446	475.2431	0.0016	0	D.EFKA.D<br>+ Glu->pyro-Glu (N-term E)
149	153	591.2799	590.2726	590.27	0.0026	1	D.EFKAD.E<br>+ Glu->pyro-Glu (N-term E)
273	277	547.2675	546.2602	546.2598	0.0005	0	D.LLECA.D<br>+ Dehydro (C)
273	277	548.2758	547.2685	547.2676	0.001	0	D.LLECA.D
283	288	710.3927	709.3854	709.3833	0.0022	0	D.LAKYIC.D
293	302	1109.5376	1108.5303	1108.5257	0.0047	0	D.TISSKLKECC.D<br>+ 2 Dehydro (C)
304	318	1723.9457	1722.9384	1722.9338	0.0046	0	D.KPLLEKSHCIAEVEK.D
320	330	1135.6415	1134.6342	1134.6284	0.0058	0	D.AIPENLPPLTA.D
320	334	1597.8044	1596.7971	1596.8035	- 0.0064	2	D.AIPENLPPLTADFAE.D
338	346	1082.5367	1081.5294	1081.5226	0.0068	0	D.VCKNYQEAK.D
389	404	1857.9759	1856.9686	1856.9607	0.0079	2	D.PHACYSTVFDKLKHLV.D
399	404	737.4991	736.4918	736.4959	- 0.0041	0	D.KLKHLV.D
399	405	852.529	851.5217	851.5229	- 0.0011	1	D.KLKHLVD.E
475	516	4757.4735	4756.4662	4756.4689	- 0.0027	0	D.YLSLILNRLCVLHEKTPVSEKVTK CCTESLVNRRPCFSALTP.D<br>+ 4 Dehydro (C)
475	517	4872.4878	4871.4805	4871.4959		1	D.YLSLILNRLCVLHEKTPVSEKVTK

					-<br>0.0153		CCTESLVNRRPCFSALTPD.E<br>+ 4 Dehydro (C)
518	525	936.4826	935.4753	935.4753	0.0001	0	D.ETYVPKAF.D<br>+ Glu->pyro-Glu (N-term E)
527	534	974.5112	973.5039	973.5022	0.0018	0	D.EKLFTFHA.D<br>+ Glu->pyro-Glu (N-term E)
536	540	545.2888	544.2815	544.2805	0.001	0	D.ICTLP.D<br>+ Dehydro (C)
536	540	546.2987	545.2914	545.2883	0.0031	0	D.ICTLP.D
580	585	610.1839	609.1766	609.2251	-<br>0.0484	1	D.KCCAAD.D

Table S3. MASCOT searching results of 5-min "one-step" BSA high-temperature hydrolysis mixed with DTT.

Start	End	Observed	Mr(expt)	Mr(calc)	Delta	M	Peptide
26	36	1353.7084	1352.7011	1352.7313	-0.0302	0	D.THKSEIAHRFK.D
26	37	1468.7671	1467.7598	1467.7582	0.0016	1	D.THKSEIAHRFKD.L
62	79	2013.0566	2012.0493	2012.0401	0.0092	0	D.EHVKLVNELTEFAKTCVA.D
							+ Glu->pyro-Glu (N-term E)
81	95	1597.7598	1596.7525	1596.7355	0.017	0	D.ESHAGCEKSLHTLFG.D
							+ Glu->pyro-Glu (N-term E)
97	109	1450.7464	1449.7391	1449.7286	0.0105	0	D.ELCKVASLRETYG.D
							+ Glu->pyro-Glu (N-term E)
111	130	2394.9527	2393.9454	2393.9861	-0.0407	2	D.MADCCEKQEPERNECFLSHK.D
							+ 2 Dehydro (C)
114	130	2077.9108	2076.9035	2076.8815	0.022	0	D.CCEKQEPERNECFLSHK.D
							+ 2 Dehydro (C)
136	141	695.4846	694.4773	694.4741	0.0032	0	D.LPKLKP.D
143	147	546.2301	545.2228	545.2394	-0.0165	0	D.PNTLC.D
							+ Dehydro (C)
143	152	1137.5051	1136.4978	1136.5172	-0.0194	2	D.PNTLCDEFKA.D
143	152	1137.5421	1136.5348	1136.5172	0.0176	2	D.PNTLCDEFKA.D
148	153	724.3238	723.3165	723.3075	0.009	2	C.DEFKAD.E
154	195	5208.5402	5207.5329	5207.489	0.0439	0	D.EKKFWGKYLYEIARRHPYFYAPELLYYANKYNGVFQECCQAE.D
							+ 2 Dehydro (C); Glu->pyro-Glu (N-term E)
154	196	5341.5553	5340.548	5340.5266	0.0215	1	D.EKKFWGKYLYEIARRHPYFYAPELLYYANKYNGVFQECCQAED.K
							+ 2 Dehydro (C)
261	271	1252.5897	1251.5824	1251.5853	-0.0028	0	D.LTKVHKECCHG.D
							+ 2 Dehydro (C)
261	277	1897.9039	1896.8966	1896.877	0.0196	2	D.LTKVHKECCHGDLLECA.D
							+ Dehydro (C)
273	277	547.2653	546.258	546.2598	-0.0017	0	D.LLECA.D
							+ Dehydro (C)
273	279	778.3427	777.3354	777.3215	0.014	2	D.LLECADD.R
279	282	476.2251	475.2178	475.2027	0.0152	2	D.DRAD.L

283	288	710.3935	709.3862	709.3833	0.003	0	D.LAKYIC.D
293	318	2930.4849	2929.4776	2929.4837	-0.006	2	D.TISSKLKECCDKPLLEKSHCIAEVEK.D
							+ Dehydro (C)
303	319	1953.994	1952.9867	1952.9877	-0.001	2	C.DKPLLEKSHCIAEVEKD.A
304	318	1722.9467	1721.9394	1721.926	0.0134	0	D.KPLLEKSHCIAEVEK.D
							+ Dehydro (C)
320	330	1135.6436	1134.6363	1134.6284	0.0079	0	D.AIPENLPPLTA.D
332	335	481.1964	480.1891	480.1856	0.0035	1	D.FAED.K
332	336	609.2925	608.2852	608.2806	0.0046	2	D.FAEDK.D
336	346	1325.6074	1324.6001	1324.6445	-0.0444	2	D.KDVCKNYQEAK.D
338	346	1081.5482	1080.5409	1080.5148	0.0261	0	D.VCKNYQEAK.D
							+ Dehydro (C)
338	347	1197.5725	1196.5652	1196.5495	0.0157	1	D.VCKNYQEAKD.A
338	386	5730.7902	5729.7829	5729.787	-0.0041	2	D.VCKNYQEAKDAFLGSFLYEYSRRHPEYAVSVLLRLAKEYEATLEECCAK.D
							+ 3 Dehydro (C)
348	386	4553.3119	4552.3046	4552.2559	0.0488	0	D.AFLGSFLYEYSRRHPEYAVSVLLRLAKEYEATLEECCAK.D
							+ 2 Dehydro (C)
348	387	4668.3299	4667.3226	4667.2828	0.0398	1	D.AFLGSFLYEYSRRHPEYAVSVLLRLAKEYEATLEECCAKD.D
							+ 2 Dehydro (C)
389	397	1024.419	1023.4117	1023.4484	-0.0367	0	D.PHACYSTVF.D
389	404	1857.9717	1856.9644	1856.9607	0.0037	2	D.PHACYSTVFDKLKHLV.D
399	404	737.5068	736.4995	736.4959	0.0036	0	D.KLKHLV.D
406	415	1167.5848	1166.5775	1166.5628	0.0147	0	D.EPQNLIKQNC.D
							+ Dehydro (C); Glu->pyro-Glu (N-term E)
406	416	1300.6406	1299.6333	1299.6003	0.033	1	D.EPQNLIKQNCD.Q
							+ Dehydro (C)
417	473	6428.292	6427.2847	6427.2464	0.0384	0	D.QFEKLGEYGFQNALIVRYTRKVPQVSTPTLVEVSRSLGKVGTRCCTKPESERMPCTE.D
							+ Dehydro (C); Gln->pyro-Glu (N-term Q)
417	474	6544.2892	6543.2819	6543.2811	0.0008	1	D.QFEKLGEYGFQNALIVRYTRKVPQVSTPTLVEVSRSLGKVGTRCCTKPESERMPCTED.Y

							+ Gln->pyro-Glu (N-term Q)
475	516	4759.4943	4758.487	4758.4845	0.0025	0	D.YLSLILNRLCVLHEKTPVSEK<br>VTKCCTESLVNRRPCFSALTP.D
							+ 2 Dehydro (C)
475	517	4873.5564	4872.5491	4872.5037	0.0454	1	D.YLSLILNRLCVLHEKTPVSEK<br>VTKCCTESLVNRRPCFSALTPD.<br>E
							+ 3 Dehydro (C)
475	525	5811.0175	5810.0102	5809.9946	0.0157	2	D.YLSLILNRLCVLHEKTPVSEK<br>VTKCCTESLVNRRPCFSALTPD<br>ETYVPKAF.D
							+ Dehydro (C)
527	534	992.5367	991.5294	991.5127	0.0167	0	D.EKLFTFHA.D
580	584	493.1999	492.1926	492.1825	0.0101	0	D.KCCAA.D
							+ 2 Dehydro (C)
580	585	610.1861	609.1788	609.2251	-0.0462	1	D.KCCAAD.D
587	607	2161.1609	2160.1536	2160.1375	0.0162	0	D.KEACFAVEGPKLVVSTQTAL<br>A.-
							+ Dehydro (C)

Table S4. MASCOT searching results of 2-min BSA high-temperature hydrolysis reduction after incubation with DTT for 1 h.

Start	End	Observed	Mr(expt)	Mr(calc)	Delta	M	Peptide
81	95	1596.7644	1595.7571	1595.7277	0.0295	0	D.ESHAGCEKSLHTLFG.D
							+ Dehydro (C); Glu->pyro-Glu (N-term E)
81	96	1711.7738	1710.7665	1710.7546	0.0119	1	D.ESHAGCEKSLHTLFGD.E
							+ Dehydro (C); Glu->pyro-Glu (N-term E)
97	109	1450.744	1449.7367	1449.7286	0.0081	0	D.ELCKVASLRETYG.D
							+ Glu->pyro-Glu (N-term E)
97	109	1467.7617	1466.7544	1466.7313	0.0231	0	D.ELCKVASLRETYG.D
							+ Dehydro (C)
97	110	1564.7475	1563.7402	1563.7477	-0.0075	1	D.ELCKVASLRETYGD.M
							+ Dehydro (C); Glu->pyro-Glu (N-term E)
97	112	1767.8435	1766.8362	1766.8331	0.0031	2	D.ELCKVASLRETYGDMA.D
							+ Glu->pyro-Glu (N-term E)
133	141	994.5536	993.5463	993.5859	-0.0395	2	D.SPDLPKLKP.D
143	147	546.204	545.1967	545.2394	-0.0426	0	D.PNTLC.D
							+ Dehydro (C)
143	152	1137.54	1136.5327	1136.5172	0.0155	2	D.PNTLCDEFKA.D
154	195	5209.5264	5208.5191	5208.4969	0.0223	0	D.EKKFWGKYLYEIARRHPYFYAPELLYYANKYNGVFQECCQAE.D
							+ Dehydro (C); Glu->pyro-Glu (N-term E)
279	282	476.1695	475.1622	475.2027	-0.0404	2	D.DRAD.L
280	288	1051.5376	1050.5303	1050.5406	-0.0103	2	D.RADLAKYIC.D
							+ Dehydro (C)
303	319	1952.9894	1951.9821	1951.9799	0.0022	2	C.DKPLLEKSHCIAEVEKD.A
							+ Dehydro (C)
304	318	1722.9434	1721.9361	1721.926	0.0101	0	D.KPLLEKSHCIAEVEK.D
							+ Dehydro (C)
320	330	1135.6433	1134.636	1134.6284	0.0076	0	D.AIPENLPPLTA.D

332	335	481.2038	480.1965	480.1856	0.0109	1	D.FAED.K
348	386	4553.3045	4552.2972	4552.2559	0.0414	0	D.AFLGSFLYEYSRRHPEY AVSVLLRLAKEYEATLEEC CAK.D
							+ 2 Dehydro (C)
348	387	4668.3334	4667.3261	4667.2828	0.0433	1	D.AFLGSFLYEYSRRHPEY AVSVLLRLAKEYEATLEEC CAKD.D
							+ 2 Dehydro (C)
389	404	1857.9675	1856.9602	1856.9607	- 0.0005	2	D.PHACYSTVFDKLKHLV. D
406	415	1185.5854	1184.5781	1184.5734	0.0048	0	D.EPQNLIKQNC.D
							+ Dehydro (C)
406	416	1300.6401	1299.6328	1299.6003	0.0325	1	D.EPQNLIKQNCD.Q
							+ Dehydro (C)
417	473	6429.3023	6428.295	6428.2542	0.0409	0	D.QFEKLGEYGFQNALIVR YTRKVPQVSTPTLVEVSRS LGKVGTRCCTKPESERMP CTE.D
							+ Gln->pyro-Glu (N-term Q)
475	516	4759.4982	4758.4909	4758.4845	0.0064	0	D.YLSLILNRLCVLHEKTPV SEKVTKCCTESLVNRRPCF SALTP.D
							+ 2 Dehydro (C)
475	517	4875.5507	4874.5434	4874.5193	0.0241	1	D.YLSLILNRLCVLHEKTPV SEKVTKCCTESLVNRRPCF SALTPD.E
							+ Dehydro (C)
518	534	2042.9821	2041.9748	2042.0149	- 0.0401	2	D.ETYVPKAFDEKLFTFHA. D
536	540	545.2702	544.2629	544.2805	- 0.0176	0	D.ICTLP.D
							+ Dehydro (C)
536	541	660.3017	659.2944	659.3074	-0.013	1	D.ICTLPD.T
							+ Dehydro (C)
542	584	4886.5631	4885.5558	4885.5908	- 0.0349	2	D.TEKQIKKQTALVELLKH KPKATEEQLKTVMENFVA FVDKCCAA.D
							+ 2 Dehydro (C)
580	585	610.1853	609.178	609.2251	-0.047	1	D.KCCAAD.D
587	607	2162.1598	2161.1525	2161.1453	0.0072	0	

Table S5. Observed hydrolyzed peptides of HRP which sequence according to UniProtKB - P00433. N* denotes glycosylated Asn.

No.	Calculated. M. W.	Observed Ions	Position	Sequence
1	2646.51	883.49(3+),<br>662.88(4+)	100-124	LLTIAAQQSVTLAGGPSWRVPLGRR
2*	2590.29	932.11(3+),<br>1107.84(3+),<br>1161.85(3+),<br>1205.86(3+)	259-281	TIPLVRSFAN*STQTFFNAFVEAM
3	1924.99	963.98(2+)	231-246	NKYYVNLEEQKGLIQS
4	1854.04	928.51(2+)	133-149	LANANLPAPFFTLPQLK
5	1529.89	510.98(3+),<br>765.94(2+)	30-42	PRIAASILRLHFH
6	1419.73	710.86(2+)	67-80	AFGNANSARGFPVI
7*	1280.65	641.33(2+),<br>742.87(2+),<br>844.41(2+),<br>925.43(2+),<br>1006.46(2+),<br>1087.48(2+),<br>1153.51(2+),	183-193	RLYNFSN*TGLP
8*	1235.64	1064.96(2+),<br>1130.98(2+)	151-161	SFRNVGLN*RSS
9*	1092.51	802.37(2+),<br>903.91(2+),	248-257	QELFSSPN*AT
10*	1082.54	907.40(2+),<br>988.42(2+),<br>1054.44(2+)	57-65	N*TTSFRTEK
11	930.51	931.50(1+)	21-28	TIVNELRS
12	846.50	847.49(1+)	223-229	LRTPTIF
13	677.37	678.38(1+)	126-131	SLQAFL·
14	639.21	640.22(1+)	44-49	CFVNGC (with an internal disulfide bond)<br>|_______|
15	515.33	516.35(1+)	51-55	ASILL

References:

1. B. Smith and J. Walker, Humana Press Inc.: New Your, 2002.
2. A. Li, R. C. Sowder, L. E. Henderson, S. P. Moore, D. J. Garfinkel and R. J. Fisher, *Anal. Chem.*, 2001, **73**, 5395-5402.
